# Outcomes following duodenectomy in patients with familial adenomatous polyposis

**DOI:** 10.1055/a-2298-0038

**Published:** 2024-05-03

**Authors:** Arthur S. Aelvoet, Isabel Martin, James Cockburn, Cherryl Cabalit, Victoria Cuthill, Duncan Spalding, Olivier Busch, Barbara A.J. Bastiaansen, Susan K. Clark, Evelien Dekker, Andrew Latchford

**Affiliations:** 1 1209 Department of Gastroenterology and Hepatology, Amsterdam UMC, Amsterdam, the Netherlands; 2 571143 Cancer Center Amsterdam, Amsterdam, the Netherlands; 3 571165 Amsterdam Gastroenterology Endocrinology Metabolism, Amsterdam, the Netherlands; 4 105692 Polyposis Registry, St Mark's Hospital, Harrow, United Kingdom; 5 4615 Department of Surgery and Cancer, Imperial College, London, United Kingdom Ireland; 6 1209 Department of Surgery, Amsterdam UMC location, Amsterdam, the Netherlands

**Keywords:** Familial adenomatous polyposis, duodenal surgery, endoscopic surveillance, gastric neoplasia, jejunal neoplasia

## Abstract

**Background and study aims** Some patients with familial adenomatous polyposis (FAP) and extensive duodenal polyposis or cancer require total duodenectomy. Regular postoperative endoscopic surveillance of the remaining jejunum and stomach is recommended, but little is known about the outcomes after this surgery.

**Patients and methods** Patients with FAP who underwent either pancreatoduodenectomy (PD) or pancreas-preserving total duodenectomy (PPTD) were identified at two expert centers. Data about postoperative endoscopic surveillance outcomes were collected, as well as survival outcomes.

**Results** Overall, 119 patients (50% female) underwent duodenectomy (86 PD and 33 PPTD); 100 for benign duodenal polyposis and 19 for duodenal or ampullary cancer. Details of postoperative endoscopic surveillance were available for 88 patients (74%). During a median follow-up of 106 months, 36 patients (41%) were diagnosed with jejunal adenomas after duodenectomy, with a significantly higher proportion in patients who underwent PPTD compared with patients who underwent PD (log-rank, *P* < 0.01). Two patients developed jejunal cancer (2%). Twenty-six patients (30%) were diagnosed with a total of 66 gastric adenomas, of which 61% were located in the fundus/body and 39% in the antrum. Five patients (6%) developed gastric cancer after a median of 15 years (range 6–23 years), all but one within carpeting fundic gland polyposis. Patients who underwent surgery for cancer had worse survival than patients with benign disease and all but one patient with postoperative gastric/jejunal cancer died.

**Conclusions** After duodenectomy in FAP, a considerable risk of developing adenomas and cancer in the stomach and jejunum exists with poor cancer prognosis, highlighting the need for close postoperative endoscopic surveillance.

## Introduction

By performing colectomy and lifelong endoscopic surveillance, lower gastrointestinal polyposis can be safely managed in the majority of patients with familial adenomatous polyposis (FAP), whereas upper gastrointestinal disease is becoming an increasingly important cause of morbidity and mortality 1 2 3 . For patients with FAP, upper gastrointestinal endoscopic surveillance is recommended, beginning at age 25 years. Nearly all FAP patients develop duodenal adenomas, predominantly around the ampulla of Vater, and historic data report a lifetime risk of duodenal cancer of 5% to 10% 4 5 6 . Guidelines advise endoscopic resection of duodenal and ampullary adenomas > 10 mm in the hope that this will prevent cancer and either defer or obviate the need for major resection surgery 7 . A more aggressive technique was recently introduced, referred to as intensive downstaging polypectomy (IDP), with removal of high numbers of small duodenal adenomas 8 ; however, long-term data about effectiveness of this approach are lacking. Patients with extensive duodenal polyposis not amenable to endoscopic management or those who have duodenal or ampullary cancer require total duodenectomy, either by pancreatoduodenectomy (PD) or pancreas-preserving total duodenectomy (PPTD).

PD and PPTD are both associated with substantial morbidity, primarily due to high rates of pancreatic fistulae. Data underscore the need for ongoing postoperative surveillance. In particular, there are reports that in 59% to 81% of patients, adenomas are detected in the remaining jejunal loops, requiring an additional jejunal resection in 3% to 15% 9 10 . In these two largest cohorts, no jejunal cancers were observed during endoscopic surveillance. In addition, there is a continued need for gastric surveillance, with the emergence of gastric adenomas and cancers becoming an increasingly described clinical issue in FAP in the Western world 11 12 .

To our knowledge, limited data have been published about risk of developing gastric adenomas and cancer after total duodenectomy. In a recent study, after PPTD, two of 47 patients (4%) patients developed high-grade dysplasia in the stomach and two of 47 patients (4%) developed gastric cancer 13 . Bile reflux into the stomach may be an important contributor to development of gastric dysplasia, and therefore, it may be predictable that the risk of gastric adenoma/cancer would increase following resection of the pylorus at duodenectomy.

We aimed to evaluate long-term outcomes in patients with FAP after total duodenectomy in a large cohort in two expert centers.

## Patients and methods

We conducted a retrospective review of all patients with FAP who underwent total duodenectomy either by PD or PPTD. We studied gastric and jejunal adenoma development and causes of postoperative death. Data were extracted from two prospectively maintained databases, at St Mark’s Hospital (London, UK) and Amsterdam UMC (Amsterdam, the Netherlands), both tertiary referral centers for FAP. The study was approved by the Institutional Review Board of both centers by September 17, 2020 and informed consent was only required and obtained from patients at Amsterdam UMC.

Pancreatoduodenectomy was performed in case of cancer in a traditional fashion and reconstruction was accomplished by constructing a long isolated afferent jejunal limb (approximately 50 cm) with hepaticojejunostomy and pancreatojejunostomy; a subset of patients underwent pancreatogastrostomy. PPTD, only performed in patients for whom there was no suspicion of cancer, included dissection of the duodenum from the pancreas and construction of a short isolated afferent jejunal limb (approximately 30 cm) with a combined hepaticopancreatojejunostomy near the blind end of the limb; PPTD was only performed for benign disease, because it does not provide an oncologic resection. Computed tomography (CT) imaging was performed on patients with cancer for staging, and in more recent years, magnetic resonance imaging also was performed to assess for presence of a pancreas divisum.

Endoscopic surveillance was performed on dedicated endoscopy lists by endoscopists experienced with FAP, using gastroscopes or pediatric colonoscopes. The presence of jejunal polyps was not assessed systematically during preoperative endoscopy, given the historical nature of this cohort. In a subset of patients in whom extensive jejunal polyposis was identified, endoscopy during surgery was performed to choose the transection plane. Postoperatively, both reconstructed jejunal limbs and the stomach were assessed using high-definition white light endoscopy and dye-based or virtual chromoendoscopy at the discretion of the endoscopist. Surveillance intervals were based on the so-called neo-Spigelman stage (SS) and gastric appearance 14 .

Collected data included baseline characteristics for demographics, *APC* mutation, preoperative SS, history of gastric adenomas, type of total duodenectomy (PD or PPTD), pyloric preservation, and type of reconstruction. Data on postoperative outcomes included number, size, location and grade of dysplasia of gastric and jejunal adenomas, presence of gastric and jejunal cancer, number of fundic gland polyps, gastric and jejunal surgeries performed, and causes of death.

Continuous variables are presented as the mean with standard deviation or median with interquartile range, depending on whether the data were normally distributed or not, and compared using a *t* -test or Mann-Whitney U test. Categorical variables are presented as numbers and percentages and compared using Fisher’s exact test. Analyses were performed using SPSS 26 (IBM Corp. Released 2019. IBM SPSS Statistics for Windows, Version 26.0. Armonk, New York, United States: IBM Corp).

## Results

A total of 119 patients (50% female) with FAP underwent total duodenectomy between 1981 and 2021; 86 underwent PD and 33 underwent PPTD. Baseline characteristics are presented in Table 1 . Nineteen patients underwent PD for ampullary cancer (n = 13) or duodenal cancer (n = 6). Ninety-six patients underwent duodenectomy for extensive benign polyposis: 63 PD and 33 PPTD. Four patients underwent combined gastrectomy and duodenectomy (2 PD and 2 PPTD) for gastric cancer (n = 3) or severe gastric polyposis (n = 1) and benign duodenal polyposis. Two patients (2%) underwent PD for what was thought to be benign duodenal polyposis, which unexpectedly turned out to be cancer in the resection specimen. Of the patients who underwent surgery for benign duodenal polyposis, four (5%) had SS stage II, 18 (21%) SS stage III, and 62 (74%) SS stage IV. Within the PPTD group, 18% had SS stage III and 82% SS stage IV. Within the PD group, 8% had SS stage II, 24% had SS stage III, and 69% had SS stage IV. The pylorus was preserved in 22 patients (19%). Eleven patients (9%) had been diagnosed with one or more gastric adenomas before they underwent duodenectomy.

**Table:** **Table 1** Patient characteristics.

	119 FAP patients
Female sex, n (%)	59 (50%)
Proven pathogenic germline variant in *APC*	109 (92%)
History of (procto)colectomy	119 (100%)
Age at duodenectomy (median and IQR)	48 (40–59)
Type of duodenectomy	
Pancreatoduodenectomy	86 (72%)
Pancreas-preserving total duodenectomy	33 (28%)
Combined duodenectomy and gastrectomy	4 (3%)
Pylorus preservation	22 (19%)
Indication for duodenectomy	
Severe duodenal polyposis	100 (84%)
Duodenal cancer	6 (5%)
Ampullary cancer	13 (11%)
Preoperative Spigelman stage available*	84 (84%)
Stage II	4 (5%)
Stage III	18 (21%)
Stage IV	62 (74%)
Gastric adenoma previous to duodenectomy	9 (8%)
*For patients that underwent surgery for benign polyposis. FAP, familial adenomatous polyposis; IQR, interquartile range.	

**Fig. 1:**
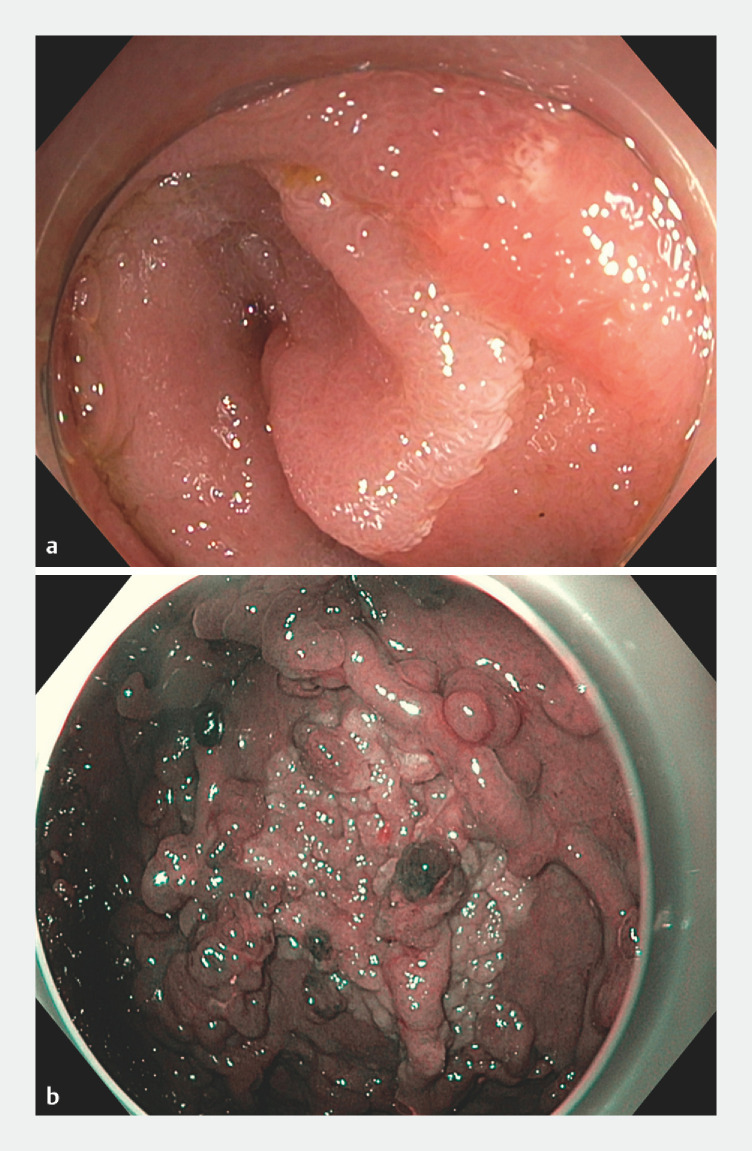
Endoscopic images of **a** jejunal and **b** gastric adenomas after duodenectomy.

In 88 of 119 patients, one or more postoperative surveillance endoscopies were performed at our centers. Median duration of endoscopic follow-up was 106 months (interquartile range [IQR] 33–167). Fig. 1 shows the endoscopic appearance of adenomas detected after duodenectomy.

### Jejunal outcomes

**Fig. 2:**
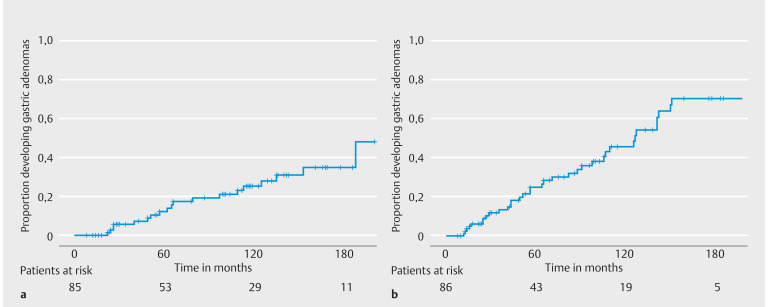
Proportion of patients diagnosed with **a** gastric and **b** jejunal adenomas.

**Fig. 3:**
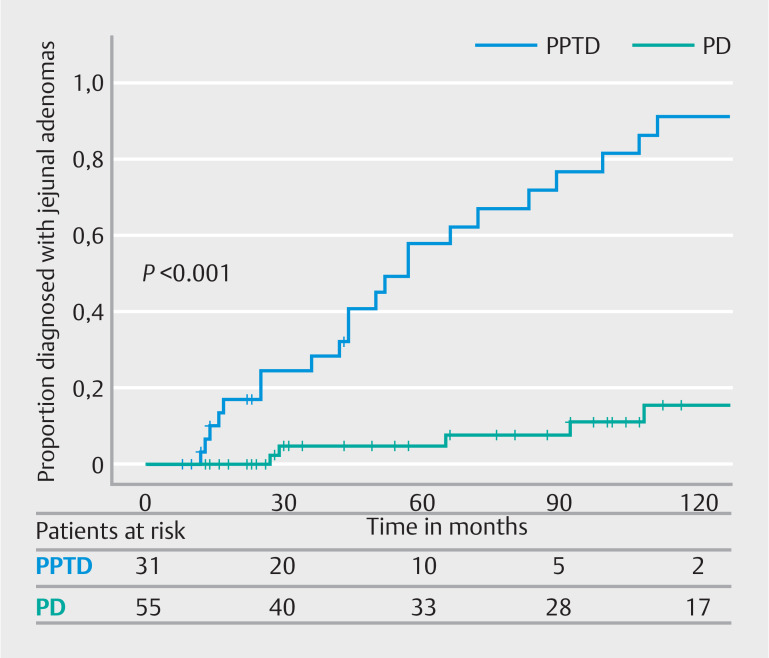
Proportion of patients diagnosed with jejunal adenomas after PPTD versus PD.

Table 2 summarizes findings from postoperative endoscopic follow-up. Thirty-six patients (41%) were diagnosed with a total of 501 jejunal adenomas after duodenectomy, of which 261 (52%) were located in the isolated afferent loop and 237 (48%) in the efferent loop. Median size of the largest adenoma detected was 16 mm (IQR 4–40). High-grade dysplasia in jejunal polyps was found in three patients (2.5%). Fig. 2 shows the proportion of patients who developed jejunal adenomas over time. There was a significant difference in detection of jejunal adenomas between patients who underwent PD and patients who underwent PPTD. At 5 and 10 years after duodenectomy, 58% vs 5% and 90% vs 15% had one or more jejunal adenomas after PPTD and PD, respectively (log rank, *P* < 0.01) ( Fig. 3 ).

**Table:** **Table 2** Findings during postoperative endoscopic follow-up.

	119 FAP patients
Duration of endoscopic follow-up (months) (median and IQR)	106 (33–167)
Number of endoscopies (median and IQR)	6 (2–11)
**Follow-up details available for the jejunum**	88 (74%)
Jejunal adenoma	36 (41%)
Total number of jejunal adenomas in cohort	501
Afferent loop	261 (52%)
Efferent loop	237 (48%)
Number of jejunal adenomas, median (IQR, range)	10 (3–20)
Jejunal adenoma with high-grade dysplasia	3 (3%)
Jejunal cancer	2 (2%)
**Follow-up details available for the stomach**	87 (73%)
Gastric adenoma	26 (30%)
Number of gastric adenomas (median and range)	1 (1–17)
Total number of gastric adenomas in cohort	66
Fundus or corpus	40 (61%)
Antrum	26 (39%)
Gastric adenoma with high-grade dysplasia	4 (5%)
Gastric cancer	5 (6%)
Fundus or body	5
Antrum	0
Fundic gland polyps	77 (88%)
0	7 (10%)
1–10	12 (17%)
11–50	5 (7%)
51–100	3 (4%)
> 100	45 (63%)
FAP, familial adenomatous polyposis; IQR, interquartile range.	

Two patients developed jejunal cancer (2%) 2.6 and 12.8 years after PD. One patient had extensive polyposis and jejunal cancer in the efferent jejunal limb diagnosed at endoscopy, which was found to be inoperable at laparotomy. The other patient had a postoperative bile leak after PD, underwent three laparotomies, presented later with an abdominal wall fistula, and was found to have jejunal cancer.

Four patients (5%) underwent an additional jejunal resection due to extensive jejunal polyposis.

### Gastric outcomes

Twenty-six patients (30%) were diagnosed with a total of 66 gastric adenomas, of which 61% were located in the fundus/body and 39% in the antrum ( Table 2 ). Nineteen of these patients (73%) had carpeting fundic gland polyposis and two had a gastric adenoma before duodenectomy (8%). The proportions of patients developing gastric adenomas at 5, 10, and 15 years after duodenectomy were 12%, 25%, and 34%, respectively ( Fig. 2 ). No differences were observed when comparing procedures in which the pylorus was preserved or resected (log rank, *P* = 0.11). Of 47 patients with features of biliary reflux/gastritis, 17 (36%) developed one or more gastric adenomas (mean 2.8). Of the 40 patients without features of biliary reflux/gastritis, nine (23%) developed one or more gastric adenomas (mean 2.1) ( *P* = 0.24).

Four patients (5%) developed gastric adenomas with high-grade dysplasia (HGD), of which three were located in the fundus or body and one in the antrum with a median adenoma size of 30 mm.

Five patients (6%) developed gastric cancer after a median of 15 years (IQR 9.5–22.4) after duodenectomy at a median age of 56. The cancers developed in the fundus (n = 1) and body (n = 4), and all except one in areas with carpeting fundic gland polyposis. One patient did not undergo endoscopic surveillance between surgery and gastric cancer diagnosis 5 years after duodenectomy. The other three patients were diagnosed with gastric cancer 5 months, 16 months, 5 years, and 6 years after their last postoperative endoscopic surveillance. Four of the five patients had been diagnosed with at least one gastric adenoma (range 1–17 adenomas) before cancer diagnosis but after duodenectomy, including two patients with a gastric adenoma with HGD. Four patients died from metastatic gastric cancer and one patient, who underwent endoscopic submucosal dissection for a T1 cancer, remained alive 16 months later and currently has no signs of metastases on CT.

### Postoperative deaths

Five- and 10-year postoperative survival estimates were 92% and 86% for patients who underwent duodenectomy for benign disease and 48% and 39% for patients who underwent duodenectomy for cancer, respectively ( *P* = 0.006). Among the 10 patients who underwent duodenectomy for cancer and died, the causes of death were as follows: metastatic duodenal/ampullary cancer (n = 7), pulmonary embolism with gastric cancer (n = 1), and unknown causes (n = 2). Among the 25 patients who underwent duodenectomy for benign disease and who died, the causes of death were as follows: metastatic gastric cancer (n = 4), jejunal cancer (n = 2), ileal pouch cancer (n = 2), Kock pouch cancer (n = 1), pancreatic cancer (n = 1), ovarian cancer (n = 1), brain tumor (n = 2), metastatic disease from an unknown primary tumor (n =2), pancreatitis (n = 1), biliary sepsis (n = 1), and other not FAP or surgery related causes (n = 8).

## Discussion

In this study, we evaluated follow-up outcomes after total duodenectomy in a relatively large cohort of FAP patients. During postoperative endoscopic surveillance, 33% of the patients were diagnosed with one or more gastric adenomas and 6% with gastric cancer. In addition, 41% of the patients were diagnosed with one or more adenomas in the remaining jejunal loops. These findings underscore that even after resection of the duodenum, high-quality endoscopic surveillance remains essential and should be improved for the stomach and jejunum.

In a smaller single-center series reporting on long-term outcomes after pancreas-sparing duodenectomy, two of 47 patients (4%) developed HGD and two of 47 patients (4%) developed gastric cancer 13 . In the same series, two of 47 patients (4%) developed HGD in the jejunum and one of 47 patients (2%) developed jejunal cancer. These findings are comparable to the findings from our study.

Because adenomas and cancer in FAP often arise around the ampulla, it has been suggested that bile might be cytotoxic in these patients. Several studies in mice and humans with FAP have shown that bile exposure may play a role in development of duodenal polyps 15 . However, a randomized study showed no effect of ursodeoxycholic acid on duodenal adenoma development 16 . Little is known about the influence of bile exposure on gastric adenoma/cancer development. A longer duration of bile exposure in the stomach was observed in FAP patients with gastric polyps compared with FAP patients without polyps 17 . A recent cross-sectional study in the sporadic setting found bile reflux to be an independent risk factor for gastric adenoma and gastric cancer 18 . We did not observe a significant difference in terms of gastric adenoma development between patients with/without pylorus and patients with/without features of biliary reflux/gastritis, which might be partly because of the sample size and potentially due to absence of documentation about bile reflux/gastritis in some cases. However, 71% of gastric adenomas developed in patients with documented biliary reflux/gastritis.

There was a striking difference in jejunal adenoma detection between patients who underwent PD compared with patients who underwent PPTD. The main difference between the two techniques lies in the reconstruction, with a separate hepaticojejunostomy and pancreatojejunostomy (or pancreatogastrostomy) after PD compared with a single hepatopancreatojejunostomy after PPTD, and a longer isolated jejunal limb after PD compared with a shorter (approximately 30 cm) jejunal limb after PPTD. A short isolated limb facilitates complete endoscopic surveillance in most patients 9 , which may be less feasible when the jejunal limb is longer. Therefore, lesions may be missed, which could explain the lower rate of detection for jejunal adenomas after PD.

It is important to note that FAP patients who do not undergo duodenal surgery also may develop jejunal adenomas and even jejunal cancer 19 20 21 . Based on the data from the present study, we cannot categorically state that jejunal adenoma development is caused by duodenal surgery itself, because systematic screening of the jejunum was performed before surgery was performed only in a subset of patients. In addition, development of jejunal adenomas may just represent the natural history of polyposis, although clinically significant jejunal lesions and cancer in the absence of duodenal surgery are very uncommon. Nevertheless, the difference in detection of jejunal adenomas according to type of surgery remains striking.

One of the limitations of the present study was the retrospective design. Retrospective collection of data from endoscopy reports is prone to inaccuracy. Completeness of endoscopy, defined as reaching the end of the isolated jejunal limb, was not documented in all reports. Moreover, it is likely that information about signs of bile reflux were not well documented. Where possible, endoscopic images were assessed to collect additional data. Prospective data collection with standardized endoscopy reports would remove this limitation. Moreover, quality of endoscopic surveillance and endoscopes has improved in recent decades, and today, endoscopists treating FAP patients are more aware of the occurrence of gastric adenoma and cancer.

Considering the challenges of diagnosing gastric adenomas in FAP, especially once located within carpeting fundic gland polyposis and against the background of biliary gastritis, it seems logical that historically, they may have been missed and may even still be missed in current practice. Therefore, in this study, the incidence may have been underestimated. Also, detection of jejunal adenomas may have improved over the study period, which might have led to a higher rate of detection of jejunal adenomas during more recently performed endoscopies. Finally, selection bias was introduced in this study, whereas patients with cancer underwent PD and patients with extensive benign polyposis PD or PPTD, which might have influenced the results.

Currently, endoscopic upper gastrointestinal surveillance protocols exist only for patients who have not undergone duodenectomy 22 23 . Centers use a “neo-Spigelman stage” for jejunum assessment after surgery, which does not take into account gastric findings. Two patients in the present study had no jejunal adenomas during follow-up, which resulted in an endoscopic surveillance interval of 5 years. At their next surveillance endoscopies, they were diagnosed with gastric cancer. This underscores the importance of gastric findings when choosing the next surveillance interval. Based on data from the present study reporting on risks of developing adenomas and cancer in the jejunum and stomach after duodenectomy, the authors propose development of a different endoscopic surveillance protocol for this specific group of patients, which should be evaluated in a prospective study. There may be an opportunity to optimize endoscopic surveillance of the stomach. In the present series, gastric cancers were observed in patients who were under active surveillance in an expert center. However, we acknowledge that some of these cases may be historical and may have been detected before the increased risk of gastric dysplasia in patients with FAP was recognized. In keeping with this, Leone et al. found that of 10 gastric cancers in FAP patients treated at a single expert center, only two were identified on endoscopy 24 . Future studies should focus on what the precursor(s) of gastric cancer in FAP is/are and how to detect them during endoscopic surveillance before they progress to cancer.

## Conclusions

Although duodenectomy can treat or prevent duodenal cancer in FAP, these patients still have a considerable risk of developing jejunal and gastric adenomas and even cancer, which should be discussed with them before they undergo surgery. There is a clear need for a better understanding of the endoscopic appearance and pathophysiology of these lesions to guide surgical decision making and to improve post-duodenectomy endoscopic surveillance.
